# Scaling the productivity of laser structuring processes using picosecond laser pulses at average powers of up to 420 W to produce superhydrophobic surfaces on stainless steel AISI 316L

**DOI:** 10.1038/s41598-018-37867-y

**Published:** 2019-02-13

**Authors:** Sebastian Faas, Uwe Bielke, Rudolf Weber, Thomas Graf

**Affiliations:** Institut für Strahlwerkzeuge (IFSW), University of Stuttgart, Stuttgart, 70569 Germany

## Abstract

We investigate the approach to scale up the productivity of the laser-based generation of superhydrophobic surfaces by means of increased average laser powers to enhance the surface structuring rates. Polished surfaces (mean roughness depth *S*_*Rz*_ = 0.076 *μ*m) of stainless steel AISI 316L were processed with a laser delivering 8 ps long pulses with a constant pulse energy of 1.4 mJ at pulse repetition rates of 100 kHz or 300 kHz corresponding to average laser powers of 140 W or 420 W, respectively. When the feed rate for the corresponding pulse repetition rate is adjusted in a way to result in a similar temperature increase due to heat accumulation effects and the re-deposition of nanoparticles formed during processing is avoided, comparable surface structures with similar wetting behavior are obtained.

## Introduction

Functionalized surfaces, e.g. superhydrophobic surfaces, are expected to be commonly used e.g. in the field of industrial production, medical technology, and home appliance^1,2^. The wetting behavior of a surface is known to be related to the surface morphology as well as the surface chemistry^3–5^. The best-known example of a superhydrophobic surface in nature is the lotus leaf. When a water droplet gets in contact with it, the droplet rolls off easily. Because the water droplet carries away any resting surface contaminations, the lotus leaf is also self-cleaning^6,7^.

The surface of stainless steel samples can be hydrophilic immediately after laser structuring. Over time, the surface becomes hydrophobic and can even reach a superhydrophobic state. The time-dependency of the wetting behavior is reported in refs^8^ and^9^. The change in the wetting behavior is supposed to be caused by atmospheric contaminations e.g. hydrocarbons which adsorb at the surface^10^. This context was studied for laser-structured aluminum^11^. Since hydrocarbons are hydrophobic, they protect the underlying hydrophilic steel surface against wetting^12^. Nevertheless, the surface chemistry of the underlying substrate may influence the wetting behavior. Especially a strong surface oxidation may lead to a higher degree of wetting^13^.

Ablation products are formed during processing which may shield the upcoming laser pulse. For processing of copper this was reported in ref.^14^. For stainless steel no shielding effects can be expected when the pulse repetition rate is below 10 MHz^15^. Additionally, nanoparticles are formed during processing which may be deposited on the surface^16^. Since these nanoparticles have a spherical shape^16^, they must have been liquid at some time and therefore they are oxidized and may be mainly responsible for the strong surface oxidation reported in ref.^13^. When processing with pulse repetition rates of hundreds of kHz the temperature increase due to heat accumulation effects was found to be a dominant factor in determining the surface structure resulting from the process^17^. In ref.^18^ an analytical model was introduced which is capable to predict the process-resulting surface structure for ultrafast lasers with an average laser power of several hundreds of Watts.

Ultrafast lasers are suitable to produce ripples, micro-grooves, and spikes^19^. Different surface structures are needed to achieve different goals, such as wetting behavior, absorption, growth of bacteria, and tribological applications. The proof of principle for these applications was reported for instance in refs^16,20–24^. In view of the industrial applications, the findings in ref.^18^ serve as basis to demonstrate superhydrophobic surfaces on stainless steel AISI 316L with a ultrafast laser with several hundreds of Watts average power for high productivity. For instance in refs^24–27^ was already reported on this upcoming challenge. Processing with a high-power laser results in surface structures with similar functionality as known from the proof-of-principle studies where a low-power laser was used, when the following applies: (a) the effects of heat accumulation are used in an adequate way, (b) no shielding effects occur, and (c) the deposition of nanoparticles on the surface is avoided.

## Experimental setup

A home-build ultrafast laser operating at a wavelength of *λ* = 1030 nm with a pulse duration of *τ* = 8 ps was used^28^. The laser delivered an average power of *P* = 525 W at a pulse repetition rate of *f* = 300 kHz, which corresponds to a pulse energy of *E*_*P*_ = 1.75 mJ. The pulse repetition rate incident on the workpiece was modified by means of an acousto-optic modulator (Isomet Corporation) having a diffraction efficiency of 80% reducing the pulse energy incident at the workpiece to a constant value of 1.4 mJ. Two pulse repetition rates (*f* = 100 kHz and *f* = 300 kHz) were applied. The laser beam was linearly polarized. The laser beam was moved over the workpiece surface by means of a fast scanner system equipped with an F-Theta lens with a focal length of 340 mm. The workpiece was placed about 2.25 Rayleigh lengths further away than the focal position of the F-Theta lens resulting in a measured beam diameter on the surface of the workpiece of *d*_*b*_ = 500 *μ*m ± 20 *μ*m. The mean fluence therefore was 0.71 J/cm^2^ ± 0.06 J/cm^2^ which is about 5.5 times above the ablation threshold of 0.13 J/cm^2^ reported in ref.^29^. The polarization was set perpendicular to the scan direction in all experiments. To realize a similar number of pulses per spot ${N}_{pps}={d}_{b}\cdot f/{v}_{feed}$ a feed rate of *v*_*feed*_ = 1 m/s was used for *f* = 100 kHz and *v*_*feed*_ = 10 m/s for *f* = 300 kHz, respectively.

Mirror-polished square plates of AISI 316L with a thickness of 2 mm, a surface area of 50 × 50 mm^2^ were used in this study. The mean roughness depth was determined with a white-light interferometer to be *S*_*Rz*_ = 0.076 *μ*m (see Table 1). The surface was cleaned with acetone before processing. A square of 10 × 10 mm^2^ was processed along parallel offset lines. To ensure a homogeneous energy input on every spot in the processed square the hatching distance *d*_*h*_ between the lines was at least one quarter of the laser beam diameter resulting in four passes per spot, respectively. When the ablation products are deposited on the processed area a strong surface oxidation may be expected which influences the wetting behavior of the resulting surface^13^. To avoid a re-deposition of the ablation products a flat nozzle was therefore used to realize an airflow parallel to the surface with a volumetric flow rate of about 42 m^3^/h during processing. After processing the structured samples were stored in ambient air.

The acousto-optic modulator (AOM) was triggered by the scanner to make sure that processing only occurred in the desired area and once the desired feed rate was reached. The processed areas were examined by means of scanning electron microscopy (SEM) and a white-light interferometer. To investigate the surface chemistry the processed areas were additionally examined by energy-dispersive X-ray spectroscopy (EDS). The static contact angle (CA) was determined using a commercial measuring device (dataphysics, OCA 15EC). The dust particles were removed with a bellows before measuring the CA. All CA-measurements took place after 50 days to avoid the phase of the changing wetting behavior^8,26^.

## Predicting the Surface Morphology Produced by Laser Processing

Since the surface of a lotus leaf exhibits a double-scaled structure with periods in the range of micrometers as well as nanometers^30^, a double-scaled structure is considered to be the most promising surface morphology to reach a superhydrophobic and self-cleaning behavior of the structured material^31^. Processing stainless steel with pulse repetition rates of hundreds of kHz result in a smooth surface which is covered with ripples with a periodicity slightly below the wavelength of the laser beam when the maximum surface temperature during processing remains below *T*_*bump*_ = 607 °C^17^. When the surface temperature during processing exceeds *T*_*bump*_ the process-resulting surface is covered with micro-grooves^17^, which corresponds to the desired double-scale structure. The periodicity of the process-resulting micro-grooves increases when the surface temperature during processing is further increased^18^. When the melting temperature is exceeded during processing, the process-resulting surface can be covered with micro-holes formed by re-solidified melt^18^. The process-resulting surface structure can therefore be well predicted theoretically by considering the heat accumulation effects^18^.

The material properties required for the corresponding calculations (density *ρ* = 8000 kg/m^3^, specific heat capacity ${c}_{p}=500\,{\rm{J}}/({\rm{kg}}\cdot {\rm{K}})$, and thermal diffusivity $\kappa =3.75\cdot {10}^{-6}\,{{\rm{m}}}^{2}/s$) are known^18^. The fraction *η*_*abs*_ = 0.55 of the absorbed energy was adopted from^18^ and the fraction *η*_*Heat*_ = 0.38 of the energy remaining in the workpiece after each pulse was adopted from^17^. In the present study similar processing parameters are used as reported in^18^ and therefore 1D heat flow may also be assumed here.

**Figure 1:**
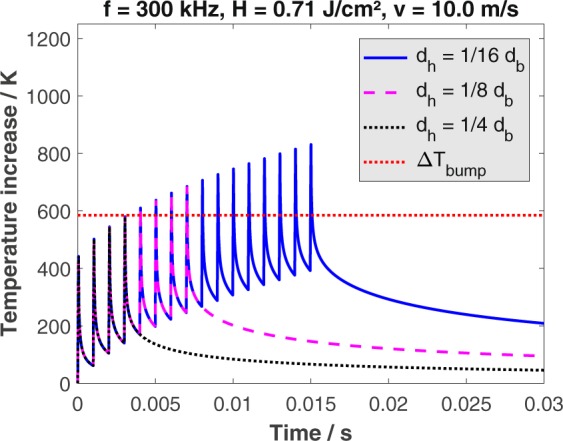
Calculated temperature increase on the surface of polished (*S*_*Rz*_ = 0.076 *μ*m) AISI 316L during processing for different number of passes (resulting from different hatching distances *d*_*h*_) as a function of time. Material parameters: *η*_*abs*_ = 0.55, *η*_*heat*_ = 0.38, *ρ* = 8000 kg/m^3^, ${c}_{p}=500\,{\rm{J}}/({\rm{kg}}\cdot {\rm{K}})$, $\kappa =3.75\cdot {10}^{-6}\,{{\rm{m}}}^{2}/s$. Process parameters: *λ* = 1030 nm, *E*_*P*_ = 1.4 mJ, *d*_*b*_ = 500 *μ*m, *H* = 0.71 J/cm^2^.

The temperature increase calculated by this model for all applied hatching distances, a constant feed rate of 10 m/s, and a pulse repetition rate of 300 kHz is shown in Fig. 1. Every peak represents the temperature increase due to heat accumulation of subsequent laser pulses within one pass^18^. Every pass results in a temperature increase of 440 K. The time between successive passes is not sufficient for the material to cool down and therefore the accumulated heat due to successive passes leads to an increased maximum temperature with every upcoming pass. The first pass leads to an accumulated heat on the surface of the workpiece of 60 K and therefore the maximum temperature increase within the second pass is 500 K. The more passes are applied the higher is the temperature increase reached with the last pass. When using a hatching distance of one quarter of the beam diameter, which corresponds to 4 passes over each point of the surface (black dotted line), the maximum induced temperature increase is 580 K. The surface resulting from this process, according to^17,18^, should therefore be covered with ripples and may show fine micro-grooves. Decreasing the hatching distance to one eighth of the beam diameter (magenta dashed line), which results in 8 processing passes on each point on the surfaces, leads to a maximum temperature increase of 685 K. The surface resulting from this process should now exhibit more pronounced micro-grooves compared to a process consisting of 4 passes per spot. Further decreasing of the hatching distance to one sixteenth of the beam diameter (blue line) results in a maximum temperature increase of 830 K. Well pronounced micro-grooves with a larger periodicity than the micro-grooves formed with 8 passes (magenta dashed line) should cover the surface resulting from this process.

**Figure 2:**
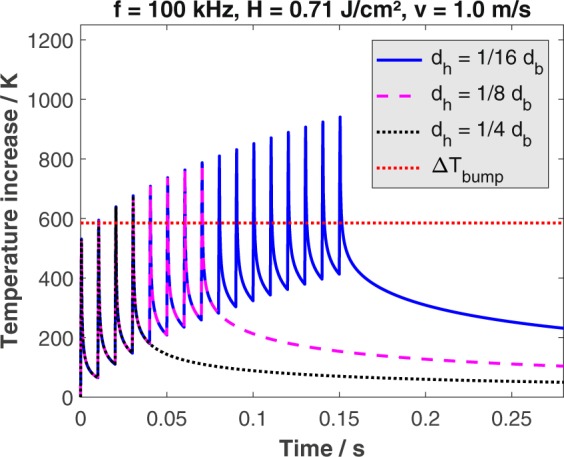
Calculated temperature increase on the surface of polished (*S*_*Rz*_ = 0.076 *μ*m) AISI 316L during processing for different number of passes (resulting from different hatching distances *d*_*h*_) as a function of time. Material parameters: *η*_*abs*_ = 0.55, *η*_*heat*_ = 0.38, *ρ* = 8000 kg/m^3^, ${c}_{p}=500\,{\rm{J}}/({\rm{kg}}\cdot {\rm{K}})$, $\kappa =3.75\cdot {10}^{-6}\,{{\rm{m}}}^{2}/s$. Process parameters: *λ* = 1030 nm, *E*_*P*_ = 1.4 mJ, *d*_*b*_ = 500 *μ*m, *H* = 0.71 J/cm^2^.

The calculated temperature increase for a feed rate of 1 m/s and a pulse repetition rate of 100 kHz is shown in Fig. 2. When using a hatching distance of one quarter of the beam diameter (black dotted line), the maximum temperature increase is 675 K. This temperature is roughly the same as obtained with *f* = 300 kHz and a hatching distance of one eighth of the beam diameter (see magenta dashed line in Fig. 1) and therefore the resulting surface should also be similar. Decreasing the hatching distance to one eighth of the beam diameter (magenta dashed line in Fig. 2) leads to a maximum temperature increase of 790 K. This temperature is roughly the same as obtained with *f* = 300 kHz and a hatching distance of one sixteenth of the beam diameter (compare blue line in Fig. 1) and therefore the resulting surface should be similar again. Further decreasing of the hatching distance to one sixteenth of the beam diameter (blue line in Fig. 2) results in a maximum temperature increase of 940 K. Micro-grooves having a larger periodicity than the micro-grooves formed with 8 passes (magenta dashed line in Fig. 2) are expected to cover the surface resulting from this process.

## Experimental Results

**Figure 3:**
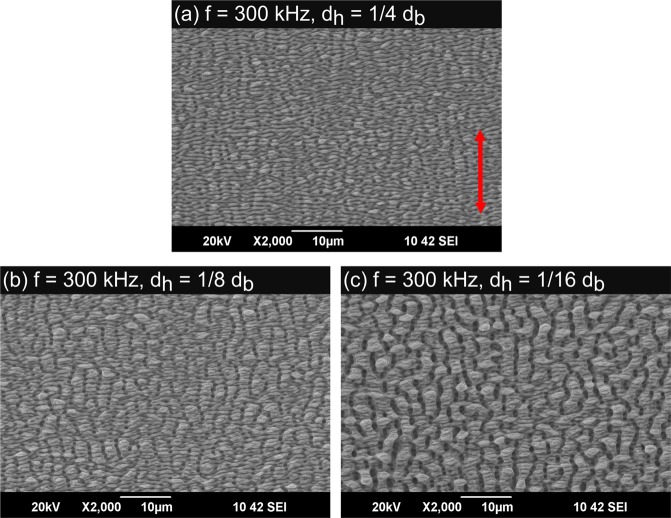
Laser induced periodic surface structures (LIPSS) on polished (*S*_*Rz*_ = 0.076 *μ*m) stainless steel AISI 316L formed with different hatching distances *d*_*h*_: (**a**) 1/4 *d*_*b*_, (**b**) 1/8 *d*_*b*_, (**c**) 1/16 *d*_*b*_. The red double arrow indicates the direction of the polarization for all applied hatching distances. Process parameters: *λ* = 1030 nm, *f* = 300 kHz, *E*_*P*_ = 1.4 mJ, *d*_*b*_ = 500 *μ*m, *H* = 0.71 J/cm^2^, *v* = 10 m/s.

Figure 3 shows laser-induced periodic surface structures (LIPSS) that were produced by applying different hatching distances during laser processing with a pulse repetition rate of 300 kHz and a feed rate of *v* = 10 m/s corresponding to the parameters used to calculate the temperature increases shown in Fig. 1. Thanks to the used cross-jet no strong covering of nanoparticles can be seen on any processed surface. Applying a hatching distance of one quarter of the beam diameter leads to a surface which is covered with ripples (Fig. 3(a)). Decreasing the hatching distance to one eighth of the beam diameter results in the formation of fine micro-grooves perpendicular to the ripples (Fig. 3(b)). Further decreasing of the hatching distance to one sixteenth of the beam diameter leads to the formation of well-pronounced micro-grooves with a periodicity of a few microns (Fig. 3(c)). Hence, the experimental results are well consistent with the above calculations as discussed with Fig. 1.

**Table 1:** Measured mean roughness depths S_Rz_ of an untreated surface and for the structured surfaces applying different hatching distances. Further parameters: *λ* = 1030 nm, *f* = 300 kHz, *d*_*b*_ = 500 *μ*m, *H* = 0.71 J/cm^2^, *v* = 10.0 m/s.

d_h_/d_b_	Mean roughness depth *S*_*Rz*_/*μ*m	Surface structure type
untreated surface	0.076 ± 0.004	polished
1/4	1.467 ± 0.035	ripples
1/8	2.088 ± 0.035	ripples/micro-grooves
1/16	3.704 ± 0.347	micro grooves

All surfaces shown in Fig. 3 and an untreated, polished surface were analyzed with a white-light interferometer (Zygo NewView 7100) to determine the mean roughness depth *S*_*Rz*_, which is an easy to measure quantity, which is correlated to the type of the surface structure. This device is capable to measure profile heights down to 1 nm with a lateral optical resolution of about 300 nm. On each surface, the measurement was repeated on 5 different places. The mean values of *S*_*Rz*_ including the corresponding standard deviations as well as the process-resulting surface structure are listed in Table 1 for all resulting surface structures shown in Fig. 3. The mean roughness depth of an untreated, polished area was 0.076 *μ*m. After processing the mean roughness depth was significantly increased, e.g. applying a hatching distance of one quarter of the beam diameter results in a mean roughness depth of 1.467 *μ*m. When the hatching distance is decreased the mean roughness depth increases further.

**Figure 4:**
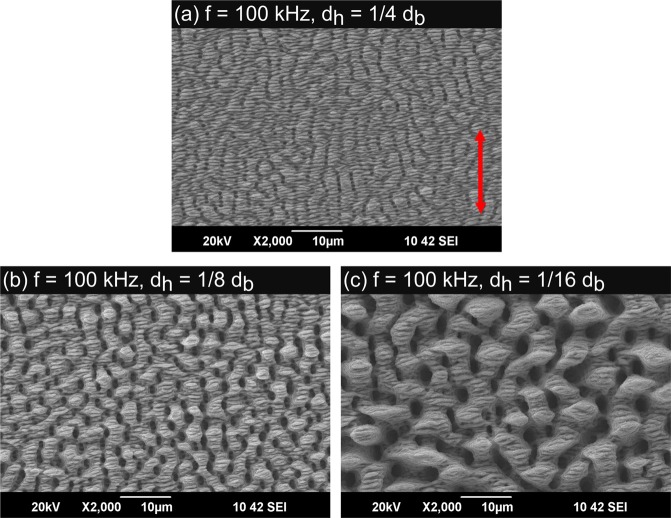
Laser induced periodic surface structures (LIPSS) on polished (*S*_*Rz*_ = 0.076 *μ*m) stainless steel AISI 316L formed with different hatching distances *d*_*h*_: (**a**) 1/4 *d*_*b*_, (**b**) 1/8 *d*_*b*_, (**c**) 1/16 *d*_*b*_. The red double arrow indicates the direction of the polarization for all applied hatching distances. Process parameters: *λ* = 1030 nm, *f* = 100 kHz, *E*_*P*_ = 1.4 mJ, *d*_*b*_ = 500 *μ*m, *H* = 0.71 J/cm^2^, *v* = 1 m/s.

The LIPSS produced with a pulse repetition rate of 100 kHz and a feed rate of *v* = 1 m/s are shown in Fig. 4. Again, no strong covering of nanoparticles can be seen on any processed surface due to the applied cross-jet. The processing parameters correspond to the ones used to calculate the temperature increases shown in Fig. 2. Applying a hatching distance of one quarter of the beam diameter leads to a surface which is covered with ripples and fine micro-grooves (Fig. 4(a)). As expected from the comparison of the calculated temperature increases above this surface is very similar to the surface shown in Fig. 3(b) since the calculated maximum temperature increase is also roughly the same for both processes. Decreasing the hatching distance to one eighth of the beam diameter results in the formation of well-pronounced micro-grooves (Fig. 4(b)). The similarity to the surface structure shown in Fig. 3(c) is again consistent with the calculated temperature increases. Further decreasing of the hatching distance to one sixteenth of the beam diameter leads to the formation of rough micro-grooves having a periodicity of up to 10 *μ*m. The experimental results again are well consistent with the calculations above (see Fig. 2).

The measured mean roughness depth *S*_*Rz*_ of the surfaces shown in Fig. 4, obtained with the white-light interferometer, are summarized in Table 2. It is seen that the mean roughness depth again increases with decreasing hatching distance. Surfaces which, according to the above calculations reached a similar maximum temperature increase during processing (300 kHz, *d*_*h*_ = 1/8*d*_*b*_, 10 m/s compared to 100 kHz, *d*_*h*_ = 1/4*d*_*b*_, 1 m/s & 300 kHz, *d*_*h*_ = 1/16*d*_*b*_, 10 m/s compared to 100 kHz, *d*_*h*_ = 1/8*d*_*b*_, 1 m/s) also exhibit a similar mean roughness depth S_Rz_ (within the uncertainties of the measurement).

**Table 2:** Measured mean roughness depths *S*_*Rz*_ of an untreated surface and for the structured surfaces applying different hatching distances. Further parameters: *λ* = 1030 nm, *f* = 100 kHz, *d*_*b*_ = 500 *μ*m, *H* = 0.71 J/cm^2^, *v* = 1.0 m/s.

*d*_*h*_/*d*_*b*_	Mean roughness depth *S*_*Rz*_/*μ*m	Surface structure type
untreated surface	0.076 ± 0.004	polished
1/4	2.178 ± 0.069	ripples/micro grooves
1/8	4.348 ± 0.243	micro-grooves
1/16	8.960 ± 0.812	rough micro grooves

## Surface Chemistry

The content of iron (Fe), chromium (Cr), nickel (Ni), manganese (Mn), carbon (C), and oxygen (O) on the investigated surfaces was determined by means of EDS. The chemical composition of every processed surface was measured on four different areas. Table 3 lists the average values of all measurements in percent per weight (ppw). The uncertainty of the listed average values is about 0.25% for all elements.

**Table 3:** Chemical composition of the structured and an untreated polished (*S*_*Rz*_ = 0.076 *μ*m) AISI 316 L surface in percent per weight (ppw) measured with EDS.

*f*/kHz	*v*/m/s	*dh*/*db*	Fe/ppw	Cr/ppw	Ni/ppw	Mn/ppw	C/ppw	O/ppw
untreated			72.18	17.41	7.54	1.24	0.95	0.02
300	10.0	1/4	71.09	16.55	7.17	2.62	0.73	0.90
300	10.0	1/8	70.94	16.61	7.11	2.71	0.76	0.92
300	10.0	1/16	71.05	17.12	6.73	2.97	0.66	0.86
100	1.0	1/4	70.61	16.47	7.19	2.75	0.78	1.50
100	1.0	1/8	70.34	17.03	6.67	3.16	0.69	1.45
100	1.0	1/16	69.13	17.75	5.96	3.64	0.61	2.20

Compared to an untreated surface all processed areas show a significant higher content of oxygen, which can be attributed to an oxidation of the processed surface by the ambient atmosphere during laser processing. After processing, the content of iron is slightly decreased which indicates the formation of an iron oxide. The content of manganese is slightly increased. Within the uncertainties of the measurement all other elements show no change due to laser machining. Within the uncertainties of the measurement all processed areas show a similar chemical composition. An additional input of oxygen by re-deposition of oxidized nanoparticles on the surface as reported on in^13^ was avoided by using a cross-jet during processing.

## Wetting Behavior and Structuring Rate

**Figure 5:**
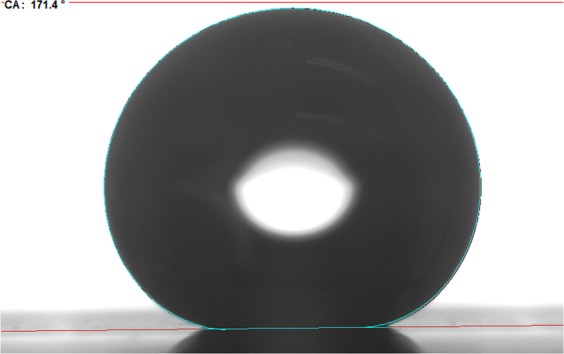
Photography of a water droplet on the double scale structure shown in Fig. 4(c). The water droplet has a static CA of 171°. Process parameters: *λ* = 1030 nm, *E*_*P*_ = 1.4 mJ, *d*_*b*_ = 500 *μ*m, *H* = 0.71 J/cm^2^, *f* = 100 kHz, *v* = 1.0 m/s, *d*_*h*_/*d*_*b*_ = 1/16.

All surface structures shown in Figs 3 and 4 were examined by a commercial measuring device to determine the static contact angle (CA). Surfaces with a double-scale structure (with micro grooves) should lead to a higher CA than surfaces covered with a mono-scale structure, e.g. ripples^8,31,32^. The measurements of the CA were performed 50 days after processing of the surfaces which is sufficient time for the structured material to reach its final wetting properties^8^. On each surface, the measurement was repeated on 5 different places. The mean value of CA including the standard deviations are listed in Table 4. Since the chemical composition of all processed areas is similar (see Table 3) one may conclude that the wetting behavior was solely influenced by the different surface morphologies. All structured areas exhibit a hydrophobic behavior. The smallest CA was measured on the surface shown in Fig. 3(a) where the surface is covered with ripples with a mono-scale structure. The largest CA was measured on the surface shown in Fig. 4(c), which is the roughest surface resulting from the process parameters used in this study, see Fig. 5. For both applied pulse repetition rates an increase of the CA can be observed when the hatching distance is decreased, which leads to a rougher surface (see Figs 3 and 4). According to^31^ a rougher surface can lead to a smaller contact area between the surface and the water droplet resulting in a larger CA. Within the uncertainties of the measurements, surfaces with a similar morphology (300 kHz, *d*_*h*_ = 1/8*d*_*b*_, 10 m/s compared to 100 kHz, *d*_*h*_ = 1/4*d*_*b*_, 1 m/s & 300 kHz, *d*_*h*_ = 1/16*d*_*b*_, 10 m/s compared to 100 kHz, *d*_*h*_ = 1/8*d*_*b*_, 1 m/s) also exhibit a similar wetting behavior.

**Table 4:** Static CA and structuring rate for all applied processing parameters.

*f*/kHz	*v*/m/s	*d*_*h*_/*d*_*b*_	Surface structuring type	Static CA/°	Structuring rate/mm^2^/s
300	10.0	1/4	ripples	136 ± 2	1250
300	10.0	1/8	ripples/micro-grooves	144 ± 4	625
300	10.0	1/16	micro-grooves	158 ± 5	312.5
100	1.0	1/4	ripples/micro-grooves	156 ± 4	125
100	1.0	1/8	micro-grooves	165 ± 3	62.5
100	1.0	1/16	rough micro-grooves	173 ± 6	31.25

Table 4 also lists the structuring rate for all processed surfaces. Structuring rates up to 312.5 mm^2^/s for micro-grooves and 1250 mm^2^/s for ripples were achieved. Due to the higher scan rate the structuring rate was larger by a factor of 5 for a pulse repetition rate of 300 kHz to achieve a similar surface morphology.

## Conclusion

Polished AISI 316L was processed with a picosecond laser providing a pulse energy of 1.4 mJ at a pulse repetition rate of 100 kHz or 300 kHz to produce superhydrophobic surfaces. We have shown that the resulting wetting behavior can reliably be predicted theoretically by considering the effects of heat accumulation. By applying different feed rates for each pulse repetition rate the heat accumulation effects were used in a way to produce similar surface structures. The chemical composition of all processed areas was similar indicating that mainly the different surface morphologies are responsible for the different wetting behavior. Surfaces with a similar morphology also showed a similar wetting behavior. Structuring rates of up to 1250 mm^2^/s were realized and the largest measured CA was 173° ± 6°. Due to heat accumulation effects the surface structuring rate increases more strongly than the pulse repetition rate.
